# Bilateral posterior crystalline lens dislocations in an otherwise healthy child

**DOI:** 10.3205/oc000077

**Published:** 2017-10-20

**Authors:** Omar A. AlShehri, Hashem Almarzouki, Badr A. Alharbi, Mohammed Alqahtani, Khaled Allam

**Affiliations:** 1 College of Medicine, King Saud Bin Abdulaziz University for Health Sciences, King Abdullah International Medical Research Center, Ministry of National Guard Health Affairs, Jeddah, Saudi Arabia; 2 King Abdullah International Medical Research Center, King Saud bin Abdulaziz University for Health Science, King Abdulaziz Medical City, National Guard Hospital, Jeddah, Saudi Arabia

**Keywords:** bilateral lens dislocation, ectopic lentis, lens displacement

## Abstract

**Introduction:** Ectopia lentis is defined as a crystalline lens displacement, either partially or completely, due to zonular abnormalities. It can be a result of trauma, hereditary ocular disease, or part of systemic diseases, like Marfan syndrome and homocystinuria.

**Case description:** We report a case of a medically free 16-year-old girl, who was referred to our hospital complaining of poor vision and a squint in both eyes since childhood. Her history included a traffic accident when she was one-year-old. She was previously diagnosed with alternating esotropia, which was treated with glasses, alternating patching, and bilateral Botox injections. On examination, she had a visual acuity of 6/7.5 with correction in the right eye and 6/6 with correction in the left eye. She had an esotropia of 60 prism diopters, which was partially corrected to 40 prism diopters for near and distance vision. Fundus examination showed myopic changes in each eye and dislocated lenses in the posterior pole at 6 o’clock. Our case was stable, so we used conservative management with contact lenses.

**Conclusion:** Bilateral posterior lens dislocation is very rare. A proper examination is important and early diagnosis can prevent serious complications, such as retinal detachment or pupillary block glaucoma.

## Introduction

Ectopia lentis is defined as an acquired or hereditary lens displaced from its natural position because of defects in the zonular filament. Karl Stellwag, an ophthalmologist from Austria, first formulated the term in 1856 and identified the lens movement within its normal space [1]. However, the prevalence of the lens dislocation is unknown. The Danish national survey carried out in 1993 showed that an estimated 6.4 per 100,000 individuals had ectopia lentis, in which most of the cases were syndromic [2]. Typically, ectopia lentis is associated with some acquired causes, such as trauma, inflammation, and hyper mature cataract. Although acquired causes are common, Williams postulated its genetic predisposition in 1875 and linked ectopia lentis to two generations in a family [3]. Genetic mutations, as in the case of Marfan syndrome (fibrilin1-gene), have been strongly associated with lens subluxation with a rate of up to 60% of the cases because of a structural defect in the ciliary zonules [4]. In the current study, we present a case of a healthy child with ectopia lentis, who had been treated for esotropia until she was referred to our center and diagnosed with bilateral posterior lens dislocation. 

## Case description

**Figure 1:**
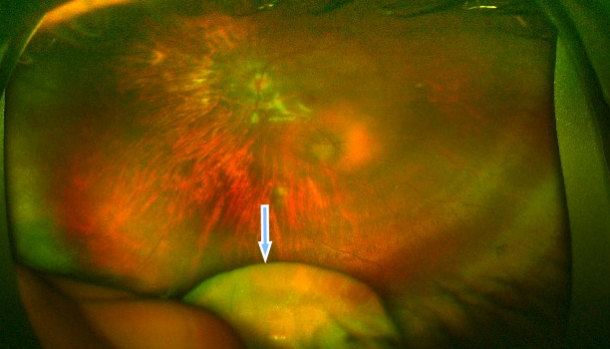
Fundus photograph of the left eye with the lens in the vitreous cavity

**Figure 2:**
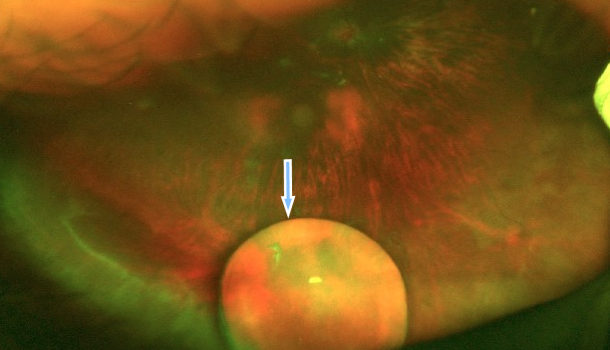
Fundus photograph of the right eye with the lens in the vitreous cavity

**Figure 3:**
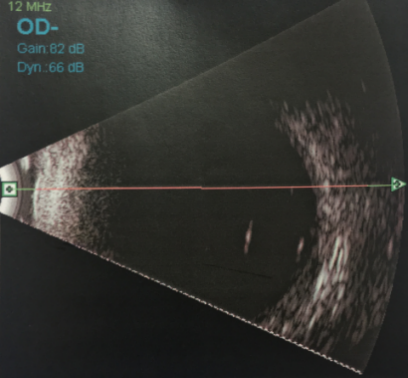
B-scan ultrasound of the right eye with vitreous opacity

**Figure 4:**
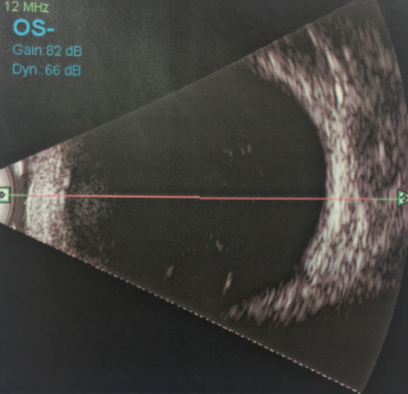
B-scan ultrasound of the left eye with vitreous opacity

A medically free 16-year-old young girl with a complaint of poor vision in both eyes since childhood was referred to our hospital from the western region of the Kingdom of Saudi Arabia. Her parents noticed that she had a squint when she was two years old, which was managed with glasses by the referring center. When the girl was one-year-old, she was involved in a motor accident, and her father provided the details of the accident that involved the child hitting her head on the windscreen leading to the breaking of the windscreen. Birth history and family history were not significant. On examination, she underwent atropine penalization refraction of +7.5–1.75x25 in the right eye and +6.75–2.25x170 in the left eye. She had a visual acuity of 6/7.5 with correction in the right eye and visual acuity of 6/6 with correction in the left eye. She had an esotropia of 60 prism diopters partially corrected to 40 prism diopters in near and distance. Esotropia was treated at first with glasses and alternating patching. Then, she underwent Botox injections bilaterally at the age of 10, and bilateral medial rectus muscle recession when she was 15 years old. Her pupils were not dilating after administering mydriatics in both eyes. Fundus examination showed bilateral myopic changes and dislocated lenses in the posterior pole at 6 o’clock. When an optos fundus photo was taken, the photo showed both lenses in the vitreous (Figure 1 (Fig. 1), Figure 2 (Fig. 2)). B-scan ultrasonography was done, which confirmed the dislocated lenses in both eyes (Figure 3 (Fig. 3), Figure 4 (Fig. 4)). In addition, an A-scan showed an axial length of 28.18 cm OD and 28.83 cm OS. Corneal topography showed no abnormalities, and systemic examination was unremarkable.

## Discussion

Lens dislocation could be a result of a high-energy trauma, hereditary ocular diseases, or parts of systemic diseases, such as Marfan syndrome and homocystinuria [5]. The clinical presentation and expected complications depend on whether it is a complete or partial dislocation, where the lens is displaced, and other associated abnormalities. In this context, anterior lens dislocation into the anterior chamber can increase the risk of acute angle closure glaucoma, while posterior dislocation, in which the lens is buried deep in the vitreous cavity, may lead to retinal detachment [6].

In this case, it was revealed that the girl suffered from bilateral posterior lens dislocation, which eventually led to a partial decrease of the refractive esotropia with the correction. The girl underwent a Botox injection of the medial rectus to alleviate the esotropia 3 years before the presentation to our center. To her father's concern, there was no improvement. The mechanism for this could be explained by a blunt head trauma that led to zonular fibers rupture with a force strong enough to rupture the anterior hyaloid phase [7]. Our case provided a history of blunt trauma to the head at one year of age. Trauma has been reported as the most common cause of crystalline lens dislocation in a study of 166 hospitalized cases [8]. However, a complete bilateral traumatic lens dislocation is very rare, and very few cases of traumatic bilateral lens dislocation have been reported in the literature. A case from Thailand was found to have an anterior lens dislocation that resulted from water splash [9]. Another case was a physically abused elderly person who suffered a posterior lens dislocation in Japan [10].

Ectopia lentis is commonly associated with high rates of genetic syndromes that could reach up to 90%, but they tend to have subluxated lenses rather than complete dislocation [11], [12]. The significance of this finding is to prevent and monitor associated life-threatening complications. Our case did not present with features that depicted any systemic genetic malformation, such as Marfan syndrome and homocystinuria. In our case, she did not suffer from any other hereditary ocular diseases, such as simple ectopia lentis or ectopia lentis et pupille. Also, family history was unremarkable in this regard. She was referred to a geneticist for counseling and further work-up.

Although spontaneous bilateral lens dislocation is rare, there have been a few cases reported. For example, there was a case of a 9-month-old infant with photophobia who was diagnosed with bilateral spherophakia and anterior lens dislocation in India [13]. Moreover, a case of a young boy from Serbia suffered from a spontaneous dislocated lens in the left eye followed by the right eye 2 years later [14]. 

The management of ectopia lentis remains a challenge for the ophthalmologists. A multidisciplinary team should be involved in managing associated systemic or genetic diseases. The goal of the treatment is to achieve good visual acuity for the prevention of amblyopia in children and other complications [6]. The primary treatment for the patients is to implement a conservative approach to address the refractive errors with the aid of eyeglasses and contact lenses. The surgical intervention should not be considered if there is no evidence of complications or failure of medical treatment [15]. However, some authors with improved efficacy and outcome of surgical techniques suggest early surgical intervention for better visual outcomes [16]. In our case, since the lenses have been dislocated for a long time with no adverse events and having achieved good visual acuity with the glasses, we preferred not to extract the lenses. Furthermore, invasive attempts to remove the lenses are associated with potential complications, such as glaucoma and retinal detachment which have been reported in the literature [17].

Finally, a special emphasis should be given to the importance of carrying out a proper physical examination and addressing all patient’s complaints, as it could lead to prevention of life threatening conditions and a better overall health outcome.

## Notes

### Competing interests

The authors declare that they have no competing interests.
